# Discriminating factors of body composition characteristics for academic performance in nursing college students: a cross-sectional study

**DOI:** 10.1186/s12912-024-01969-y

**Published:** 2024-05-03

**Authors:** Andrew Ke-Ming Lu, Shi-Yen Tsai, Ching-Yi Lin, Jeng-Long Hsieh

**Affiliations:** 1 Department of Nursing, College of Nursing, Chung Hwa University of Medical Technology, Tainan, Taiwan; 2 Healthcare Information Technology Education Center, College of Nursing, Chung Hwa University of Medical Technology, Tainan, Taiwan; 3 Department of Nursing, Kuo General Hospital, Tainan, Taiwan; 4 Library and Information Office, Chung Hwa University of Medical Technology, Tainan, Taiwan

**Keywords:** Body composition, Academic performance, Nursing college students

## Abstract

**Background:**

Poor body composition may affect health status, and better body composition is often associated with better academic performance. Nursing students face heavy academic and practical pressures, and the relationship between body composition and academic performance in this group is not fully understood.

**Methods:**

This cross-sectional observational study used de-identified student data from a university of technology in southern Taiwan to analyze the correlation between body composition characteristics and academic performance using regression models.

**Results:**

A total of 275 nursing college students were divided into four groups according to academic performance. The group with the lowest academic performance had a lower percentage of body fat (*P* < 0.05) but a higher percentage of muscle mass (*P* < 0.05) than the other three groups. Academic performance was positively correlated with percentage of body fat (*R* = 0.16, *P* < 0.01) and body age (*R* = 0.41, *P* < 0.01), but was negatively correlated with percentage of muscle mass (*R* = − 0.16, *P* < 0.01). Percentage of body fat, visceral fat area, and body age were significant discriminators of academic performance (*P* < 0.05).

**Conclusions:**

The relationship between academic performance and body composition among nursing college students is not straightforward. Contrary to our initial hypothesis, students with higher academic performance tended to have a higher percentage of body fat and a lower percentage of muscle mass. Percentage of body fat, visceral fat area, and body age were significant discriminators of academic performance, indicating that body composition should be considered an important factor in nursing education and practice.

## Introduction

Body composition is a term that refers to the relative proportions of different tissues in the body, including fat, muscle, water, and others [1]. Maintaining a healthy body composition is essential for overall good health [2]. Excessive body fat has been linked to many health problems, such as cardiovascular disease, high blood pressure, and diabetes [3]. Visceral fat, also known as intra-abdominal fat or deep abdominal fat, surrounds the organs and is stored within the abdominal cavity [4]. High levels of visceral fat are associated with an increased risk for several health problems [5]. Measuring and monitoring visceral fat area can be a useful tool in assessing health risks associated with excess visceral fat [6]. In contrast, greater muscle mass can increase the body’s metabolic rate and energy expenditure, which increases caloric burn [7], and may improve balance and stability, preventing accidental injuries such as falls and fractures [8]. A large-scale epidemiological study of body composition among Taiwanese participants found that Taiwanese people have a relatively lower body mass index (BMI) but a higher percentage of body fat than Caucasians [9]. There are also studies indicating that high obesity and BMI among young people are associated with increased mortality rates [10]. In summary, proper management of body fat and muscle mass can help prevent many health issues and promote overall health.

Several factors may affect body composition characteristics among nursing students. First, nursing students often face rigorous coursework, practice, and other study-related pressures that can lead to physical and mental fatigue [11]. Fatigued individuals may experience a lack of motivation to engage in physical activity [12], which in turn leads to weight gain and changes in body composition. Second, students may spend extended periods of time sitting in class or in front of computers to study [13], which leads to poor posture and decreased muscle mass [14]. Third, the lack of exercise due to time pressure from undertaking studies and internships may contribute to an unhealthy body composition [15]. Regular exercise is important for maintaining muscle mass, burning calories, and reducing the risk of chronic diseases [16]. The lack of exercise can lead to muscle wasting, decreased metabolism, and increased body fat [17]. Fourth, one study demonstrated that almost a third of nursing students had poor sleep habits [18]. Abnormal sleep patterns have also been linked to a decrease in lean muscle mass and an increase in body fat, which negatively affects body composition [19].

Higher BMI and body fat levels have been linked to lower academic achievements in children and adolescents [20], whereas healthy body composition has been associated with higher academic performance [21]. Similarly, overweight and obese students among adolescents aged 12–17 years have been found to achieve lower grades and experience increased missed days due to illness [22]. Furthermore, a higher percentage of body fat has been associated with lower cognitive performance and reduced memory recall abilities, which may negatively impact academic performance [23]. On the other hand, a lower percentage of muscle mass has been linked to poor cognitive function [24]. Overall, body composition impacts physical fitness, energy levels, and overall health, which in turn influence academic performance [25]. Therefore, maintaining a healthy body composition through proper nutrition and regular physical activity is likely to positively affect academic performance [26].

We were interested in exploring whether there is a correlation between students’ health by measuring body composition (including factors such as percentage of body fat, muscle mass, etc.) and their academic performance. The rationale of the study is relevant to the nursing profession, given that this study involved students in a college of nursing, one may be particularly interested in understanding the relationship between physical health as reflected in body composition and their academic success and potentially future performance as health care professionals. Further, provide educational strategies and intervention measures. Identifying discriminating factors in body composition that may influence academic performance can inform the development of targeted interventions or educational strategies. For example, if certain body composition characteristics are found to be associated with better academic performance, educational programs can be designed to promote and support these characteristics. Our hypothesis that maintaining a healthy body composition would lead to better academic performance. By examining this relationship, we aimed to provide nursing educators with a deeper understanding of the health status of their students and to offer health promotion advice to optimize academic performance. The broader goal of our research was to promote the academic and professional success of nursing students by improving their overall health and well-being.

## Methods

### Research design

We performed a cross-sectional study to investigate the association between body composition characteristics, academic performance, conduct grade, and sick leave among nursing college students. This study was correlational in that it evaluated the relationship between variables, and cross-sectional in that it collected and analyzed data from several variables at a specific point in time. Academic performance was regarded as the criterion variable, and body composition characteristics were used as discriminators.

### Data collection

The research data for this study were obtained from a medical technology university in southern Taiwan. Body composition data were obtained from the Healthcare Information Technology Education Center at the College of Nursing. Body composition data is a service provided by the College of Nursing for students to monitor their own health status. There will be a fixed time for students to do monitoring every semester. Student academic grades, conduct grades, and sick leaves records were obtained from the Office of Institutional Research database. The student’s academic grades are the average score of all academic subjects in the semester; the conduct grades are based on the basic score of 85 out of 100 set by the school, with bonus and subtraction points based on absences, rewards and punishment records; the sick leave records are the total hours of periods of leave due to illness in the semester. Students signed a data use consent form upon enrollment. All data were de-identified and approved by the Institutional Review Board (IRB). Body composition and academic data were linked by extracting academic grades, conduct grades, and sick leaves records corresponding to the semester when body composition was measured. Our data concatenation is performed through the number generated after de-identified. The research data were collected from July 2020 to July 2022 and included nursing college students aged 18 to 25 who were enrolled including the second year of the four-year system, the first year of the two-year system, and the fourth year of the five-year system. Although the systems are different, the nursing students targeted are all in the classroom learning stage and face the similar request from school work. The four-year bachelor’s degree program is designed for students who have completed senior high school; the five-year associate degree program (junior college) is designed for students who have completed junior high school; the two-year bachelor’s degree program is designed for students who have completed associate degree program. There are different ways to enter the nursing field in Taiwan. All course designs include classroom learning and clinical internships to cultivate talents in nursing and related medical fields. This study included 275 nursing students. To mitigate potential research biases, this study has implemented exclusion criteria targeting specific conditions within the dataset. These criteria include age restrictions, with participants under 18 and over 25 years old being excluded. Additionally, individuals with significant medical issues, such as cancer, severe movement disorders, and mental illnesses, have been excluded from the study cohort. The average age of these 275 students (203 females, 72 males) was 19.26 ± 1.94 years, the average height was 162.33 ± 8.67 cm, the average weight was 59.01 ± 13.81 kg, and the average BMI was 22.31 ± 4.48.

### Body composition measurements

The Body Composition Analyzer (ACCUNIQ BC710, Selvas Healthcare, Republic of Korea) uses the bioelectrical resistance method to measure and analyze the body fat and non-fat content of the body trunk and limbs. This instrument can accurately measure body fat, water content, muscle, and bone weight, which is used to understand the health status. In addition to evaluating overall body composition, the instrument also analyzes the condition of individual body parts and provides complete evaluation results for the left/right arm, left/right leg, waist/hip ratio, upper/lower limb balance, and left/right limb balance. Body age is determined by taking into account factors such as weight, percentage of body fat, and skeletal muscle percentage. This calculation produces a reference point to evaluate whether your body age is higher or lower than your chronological age. During measurement, participants wore light clothes, removed metal objects in contact with the body, maintained the correct posture when being measured, and avoided speech and movement. We analyzed BMI, percentage of body fat (%), visceral fat area (cm^2^), percentage of muscle mass (%), and body age.

### Statistical analysis

We present continuous variables as mean ± standard error and categorical variables as proportions. Spearman rank correlation was used to investigate the relationship between variables. We classified all students into four groups based on their academic performance: Group A (academic performance ≥ 80), Group B (academic performance ≥ 70 and < 80), Group C (academic performance ≥ 60 and < 70), and Group D (academic performance < 60). Rationale for dividing the students into four groups was based on GPA grading levels at our schools. We performed ANOVA to compare the mean difference in body composition between the groups and performed multiple comparisons using a post-hoc *t*-test. Linear regression analysis was used to explore the relationship between body composition characteristics and academic performance. In addition, we used ordinal logistic regression, and computed odds ratios and their corresponding 95% confidence intervals, to explore the potential association between body composition and academic performance. The significance level was set at 95% (*P* < 0.05). Statistical analysis was performed using SAS version 9.4 (SAS Institute Inc., Cary, NC, USA).

## Results

The demographic characteristics are shown in Table 1. We included a total of 275 students in the study. All 275 students were systematically categorized into distinct groups, denoted as Group A, Group B, Group C, and Group D, based on their academic performance falling into the respective ranges of > 80, 70–79, 60–69, and < 60 out of 100. Group A includes 112 females and 24 males, with an average age of 20.02 ± 1.7 years; Group B includes 67 females and 23 males, with an average age of 18.69 ± 1.95 years; Group C includes 19 females and 18 males, with an average age of 17.95 ± 1.78 years; and Group D includes 5 females and 7 males, with an average age of 18.92 ± 1.08 years. The majority of the participants of this study were female students, and Group A was slightly older than Groups B, C, and D. Regarding academic performance, the average scores for Groups A through D were 85.37 ± 3.42, 75.25 ± 3.09, 65.72 ± 2.57, and 53.3 ± 6.1 out of 100, respectively. In terms of conduct grade, the average scores for Groups A through D were 86.6 ± 3.3, 84.68 ± 5.64, 78.04 ± 8.38, and 70.5 ± 9.74 out of 100, respectively. The four groups differed in academic performance and conduct grades, ranked in descending order from group A–D. The average numbers of sick leaves during the semester for Groups A through D were 2.87 ± 6.98, 6.86 ± 11.51, 21 ± 27.76, and 53 ± 54.54 h, respectively. Both Group C and Group D had significantly higher rates of sick leave than Group A and Group B.

**Table 1:** General characteristics of nursing college students

	Group A (*n* = 136)	Group B (*n* = 90)	Group C (*n* = 37)	Group D (*n* = 12)	
Variables	n, %	n, %	n, %	n, %	
Gender (Female)	112 (82.35%)	67 (74.44%)	19 (51.35%)	5(41.67%)	
	Mean, SD	Mean, SD	Mean, SD	Mean, SD	P-value
Academic performance	85.37 ± 3.42	75.25 ± 3.09	65.72 ± 2.57	53.3 ± 6.1	a, b, c, d, e, f
Age (Years)	20.02 ± 1.7	18.69 ± 1.95	17.95 ± 1.78	18.92 ± 1.08	a, b, c
Conduct grade	86.6 ± 3.3	84.68 ± 5.64	78.04 ± 8.38	70.5 ± 9.74	a, b, c, d, e, f
Sick leave (hours/semester)	2.87 ± 6.98	6.86 ± 11.51	21 ± 27.76	53 ± 54.54	b, c, d, e, f

Group A: academic performance ≥ 80; Group B: academic performance ≥ 70 and < 80; Group C: academic performance ≥ 60 and < 70; Group D: academic performance < 60. Lower-case letters indicate significant differences between two specific groups (*P* < 0.05)

a: A and B; b: A and C; c: A and D; d: B and C; e: B and D; f: C and D

The body composition characteristics of nursing college students are shown in Table 2. For Groups A through D, the body mass index (BMI) values were 22.24 ± 4.39, 22.19 ± 4.08, 23.49 ± 5.52, and 20.3 ± 4.39 kg/m², respectively. The percentage of body fat for these groups was 23.93 ± 7.48, 23.5 ± 7.18, 22.32 ± 9.49, and 16.32 ± 9.36%, respectively. Visceral fat area values for Groups A through D were 40.9 ± 27.03, 33.89 ± 31.15, 37.32 ± 42.3, and 39.08 ± 32.53 cm², respectively. Percentage of muscle mass values were 70.3 ± 7.41, 70.74 ± 7.13, 71.92 ± 9.4, and 77.87 ± 9.3%, respectively. Additionally, the body age for Groups A through D was 20 ± 2.01, 18.7 ± 2.06, 17.81 ± 1.52, and 18.92 ± 1.08, respectively. We compared BMI, percentage of body fat, visceral fat area, percentage of muscle mass, and body age between groups, and found that Group D had significantly lower percentage of body fat than Groups A, B, and C, and significantly higher percentage of muscle mass than Groups A, B, and C. However, Group A had a significantly higher body age than Groups B and C.

**Table 2:** Body composition characteristics of nursing college students

	Group A	Group B	Group C	Group D	*P*-value
Variables	Mean, SD	Mean, SD	Mean, SD	Mean, SD	
BMI (kg/m2)	22.24 ± 4.39	22.19 ± 4.08	23.49 ± 5.52	20.3 ± 4.39	f
Percentage of body fat (%)	23.93 ± 7.48	23.5 ± 7.18	22.32 ± 9.49	16.32 ± 9.36	c, e, f
Visceral fat area (cm^2^)	40.9 ± 27.03	33.89 ± 31.15	37.32 ± 42.3	39.08 ± 32.53	
Percentage of muscle mass (%)	70.3 ± 7.41	70.74 ± 7.13	71.92 ± 9.4	77.87 ± 9.3	c, e, f
Body age	20 ± 2.01	18.7 ± 2.06	17.81 ± 1.52	18.92 ± 1.08	a, b, d

Lower-case letters indicate significant differences between two specific groups (*P* < 0.05)

a: A and B; b: A and C; c: A and D; d: B and C; e: B and D; f: C and D

We then performed correlation analyses between body composition and academic performance, conduct grades, and amount of sick leave (Table 3). Academic performance was positively correlated with conduct grades (*R* = 0.58, *P* < 0.01) and negatively correlated with the amount of sick leave (*R* = − 0.53, *P* < 0.01). Conduct grades were also negatively correlated with the amount of sick leave (*R* = − 0.60, *P* < 0.01). Notably, academic performance was positively correlated with percentage of body fat (*R* = 0.16, *P* < 0.01) and with body age (*R* = 0.41, *P* < 0.01), but was negatively correlated with percentage of muscle mass (*R* = − 0.16, *P* < 0.01). Similarly, conduct grades were positively correlated with percentage of body fat (*R* = 0.16, *P* < 0.01) and with body age (*R* = 0.16, *P* < 0.01), but were negatively correlated with percentage of muscle mass (*R* = − 0.16, *P* < 0.01).

**Table 3:** Correlation between body composition characteristics and academic performance among nursing college students

	Academic performance		Conduct grade		Sick leave (hours/semester)		BMI		Percentage of body fat		Visceral fat area		Percentage of muscle mass		Body age	
	Corr.	P-value	Corr.	P-value	Corr.	P-value	Corr.	P-value	Corr.	P-value	Corr.	P-value	Corr.	P-value	Corr.	P-value
Academic performance	1.00		0.58	**	-0.53	**	-0.01		0.16	**	0.08		-0.16	**	0.41	**
Conduct grade	0.58	**	1.00		-0.60	**	0.05		0.16	**	0.01		-0.16	**	0.16	**
Sick leave (hours/semester)	-0.53	**	-0.60	**	1.00		-0.04		-0.09		-0.04		0.09		-0.19	**
BMI	-0.01		0.05		-0.04		1.00		0.82	**	0.72	**	-0.82	**	0.18	**
Percentage of body fat	0.16	**	0.16	**	-0.09		0.82	**	1.00		0.54	**	1.00	**	0.20	**
Visceral fat area	0.08		0.01		-0.04		0.72	**	0.54	**	1.00		-0.54	**	0.55	**
Percentage of muscle mass	-0.16	**	-0.16	**	0.09		-0.82	**	1.00	**	-0.54	**	1.00		-0.20	**
Body age	0.41	**	0.16	**	-0.19		0.18	**	0.20	**	0.55	**	-0.20	**	1.00	

** Significant correlation at *p* < 0.01. * Significant correlation at *p* < 0.05

The results of the univariate linear regression analysis performed to identify factors influencing academic performance are shown in Table 4. The adjusted R² is 25.44%. The regression model revealed that percentage of body fat (β = 0.19, *P* = 0.01) and body age (β = 1.85, *P* < 0.01) were significantly and positively associated with academic performance, while percentage of muscle mass was significantly negatively associated with academic performance (β = − 0.20, *P* < 0.01).

**Table 4:** Univariate linear regression of body composition and academic performance

Variables	Beta	SD	95% CI	*P*-value
BMI	-0.01	0.13	-0.26, 0.24	0.94
**Percentage of body fat**	**0.19**	**0.07**	**0.05, 0.33**	**0.01**
Visceral fat area	0.02	0.02	-0.01, 0.06	0.20
**Percentage of muscle mass**	**-0.20**	**0.07**	**-0.34, -0.06**	**< 0.01**
**Body age**	**1.85**	**0.25**	**1.37, 2.34**	**< 0.01**

Values in bold denote *P* < 0.05

We performed a multivariate stepwise linear regression analysis with academic performance as the dependent variable. Based on the results of univariate analyses, we included BMI, percentage of body fat, visceral fat area, and body age as independent variables. The final equation included percentage of body fat (*P* < 0.01), visceral fat area (*P* = 0.01), and body age (*P* < 0.01) as significant discriminators. The detailed results are given in Table 5.

**Table 5:** Multiple linear regression of body composition characteristics and academic performance

Variables	Beta	SD	t-value	95% CI	*P*-value
Intercept	36.75	7.72	4.76	21.56, 51.94	< 0.01
BMI	-0.48	0.26	-1.87	-0.98, 0.02	0.06
**Percentage of body fat**	**0.45**	**0.11**	**4.02**	**0.23, 0.67**	**< 0.01**
**Visceral fat area**	**-0.08**	**0.03**	**-2.5**	**-0.13, -0.02**	**0.01**
**Body age**	**2.30**	**0.31**	**7.4**	**1.69, 2.92**	**< 0.01**

Values in bold denote *P* < 0.05

Furthermore, we used multiple ordinal regression analysis to examine the factors associated with academic performance among nursing college students (Table 6). BMI, percentage of body fat, visceral fat area, and body age were included as independent variables, and academic performance as the dependent variable. Our analysis revealed that percentage of body fat (*P* < 0.01), visceral fat area (*P* = 0.03), and body age (*P* < 0.01) were significant discriminators of academic performance.

**Table 6:** Ordinal regression analysis of body composition characteristics and academic performance

Variables	Odds ratio	95% CI	*P*-value
BMI	0.91	0.80, 1.02	0.11
**Percentage of body fat**	**1.10**	**1.04, 1.16**	**< 0.01**
**Visceral fat area**	**0.98**	**0.97, 1.00**	**0.03**
**Body age**	**1.62**	**1.37, 1.93**	**< 0.01**

Group D is the control group for the odds ratio

## Discussion

Our study results contradicted our hypothesis that nursing college students with better academic performance would have better body composition. Rather we, found that academic performance was positively correlated with percentage of body fat and negatively correlated with percentage of muscle mass. Furthermore, we found that percentage of body fat, visceral fat area, and body age had significant discriminatory ability on academic performance. We also found that academic performance was positively correlated with conduct grades and negatively correlated with the amount of sick leave.

One study demonstrates a trade-off between academic performance and physical health outcomes. Students who reported spending more time studying have been reported to have worse sleep habits [27], which may affect body composition. Additionally, study time is positively correlated with body fat, and weight gain has been linked to increased academic load [28, 29]. However, the results of our study contradict those of others that show a positive correlation between academic performance and physical health outcomes [30]. For example, a study found that higher academic performance was associated with better health-related behaviors and lower obesity rates [31]. Other studies found a correlation between healthy weight and improved academic performance [32], and observed that non-obese teens have higher average academic performance than obese teens [33]. These discrepancies may arise from differences between populations, countries, educational systems, and cultures studied. In particular, our results may be partially explained by our study population, which consisted of nursing college students who face heavy academic and internship pressures. Over 80% of nurses in Taiwan graduate from technical colleges (Universities of Technology and Junior Colleges), and our research may thus represent the health condition of nursing students in Taiwan’s technical education system only.

The present study has uncovered notable associations between body composition metrics, namely percentage of body fat, visceral fat area, and body age, and academic performance among nursing students. Surprisingly, our findings reveal that nursing students exhibiting superior academic performance tend to possess higher percentage of body fat, increased visceral fat area, and an older body age. In seeking to interpret this phenomenon, it is postulated that nursing students experience elevated levels of academic rigor, coupled with substantial physical and psychological stress throughout their educational journey. Notably, high-achieving students often dedicate extensive time to scholastic endeavors, potentially impacting their overall body composition. The pervasive academic demands on nursing students, including rigorous coursework and demanding clinical experiences, may necessitate extended periods of focused study. This heightened commitment to academic pursuits, in turn, could contribute to the observed correlations with body composition metrics. The intricate interplay between academic achievement and physiological parameters suggests a complex relationship, warranting nuanced consideration. It is imperative to approach these findings with a degree of caution and maintain an open-minded perspective. The individuality inherent to each student, encompassing unique learning styles and lifestyle patterns, introduces a multitude of variables that may influence body composition outcomes. Consequently, while our study provides valuable insights into potential associations between body composition and academic performance among nursing students, further research is warranted to unravel the intricate dynamics underlying these correlations. A comprehensive exploration of diverse factors, encompassing lifestyle choices, stress management, and socio-cultural influences, is essential to elucidate the multifaceted nature of this intriguing relationship.

The relationship between physical health or body composition and academic performance may have potential implications for the field of education, several comprehensive approaches can be applied to student well-being as follows. (1) Comprehensive wellness programs and health education courses: These programs can incorporate physical activity plans, nutrition education, stress management, sleep hygiene and mental health support to educate students on the importance of maintaining a healthy lifestyle. (2) Physical Education and Active Learning: Reinforcing the importance of physical education in schools and ensuring students have regular opportunities for physical activity. Consideration should also be given to incorporating active learning strategies into the classroom. (3) Support for Healthy Nutrition: Providing healthy and nutritious food choices in school cafeterias to help students maintain optimal physical health. (4) Physical and Mental Health Services: Offering on-site physical and mental health services within educational institutions. Early identification and intervention for health problems can have a positive impact on academic outcomes. (5) Research and Ongoing Evaluation: Encouraging ongoing research to further understand the subtle links between physical health and academic performance. This holistic perspective supports the development of well-rounded individuals who will be able to meet their cognitive, emotional, and physical needs throughout their educational journey.

Our study found that Taiwanese nursing students with better academic performance intended to have a high body fat rate, low muscle mass rate, high visceral fat area and high body age. We believe those may be due to the following reasons: culture and eating habits, Taiwan’s dietary habits may include foods high in fat and sugar, which may affect body composition; Taiwan’s nursing academic environment is competitive, and nursing students may experience high academic pressure, resulting in ignoring normal diet and essential nutrition; Furthermore, the lifestyle of nursing students may be relatively busy, and they may not be able to maintain regular physical activities and exercise during the semester. All of which may lead to higher physical age, a decrease in muscle mass rate, accumulation of visceral fat as well as high body fat rate.

Understanding the body composition and academic performance of nursing students has significant clinical implications and applications, particularly in nursing education and practice. First, the body composition of nursing students can provide valuable health information, including percentage of body fat and percentage of muscle mass. These data can be used to assess health risks such as obesity and muscle weakness, and provide guidance on nursing education and lifestyle advice. Additionally, such data can be used to design nutrition and exercise programs for nursing students. Second, knowledge of the body composition of nursing students can inform nursing practice, such as assessing students’ physical load and work capacity in practical nursing work. Finally, body composition data can be used for risk assessment in actual work environments, including that of stress, fatigue, and work pressure. We will investigate the relationship between factors such as mental health indicators, exercise, drinking, and smoking habits, and other multivariable factors, on student performance. These findings will provide the guideline for the improvement of nursing education and nursing practice.

## Limitations and conclusions

Our study is subject to some limitations. First, causality cannot be determined from a cross-sectional study, and future cohort studies are therefore needed to confirm our findings. Second, many factors influence academic performance, including psychological factors, learning environment, study habits, and personal traits. Accordingly, our study can only partially explain the relationship between body composition and academic performance of nursing students. Third, the nursing college students enrolled in the present study are predominantly female, and gender differences may need to be further discussed.

In conclusion, our study found that the relationship between academic performance and body composition among nursing college students is not straightforward. Contrary to the initial hypothesis, students with higher academic performance had a higher percentage of body fat and a lower percentage of muscle mass. Additionally, the present study found that percentage of body fat, visceral fat area, and body age are significant discriminators of academic performance among nursing college students. These findings suggest that body composition should be considered an important factor in nursing education and nursing practice. Finally, the study also found that academic performance was positively correlated with conduct grades and negatively correlated with the amount of sick leave, which highlights the importance of health and wellness in academic success.

## Data Availability

The datasets used and analyzed during the current study are available from the corresponding author on reasonable request.
